# Interhospital Transport on Extracorporeal Membrane Oxygenation of Neonates—Perspective for the Future

**DOI:** 10.3389/fped.2019.00329

**Published:** 2019-08-06

**Authors:** Lars Mikael Broman

**Affiliations:** ^1^Department of Pediatric Perioperative Medicine and Intensive Care, Extracorporeal Membrane Oxygenation Centre Karolinska, Karolinska University Hospital, Stockholm, Sweden; ^2^Department of Physiology and Pharmacology, Karolinska Institutet, Stockholm, Sweden

**Keywords:** extracorporeal membrane oxygenation, neonatal, neonate, children, transport, inter-hospital, interhospital, prem

## Abstract

In recent years the number of extracorporeal membrane oxygenation (ECMO) cases in neonates has been relatively constant. Future expansion lays in new indications for treatment. Regionalization to high-volume ECMO centers allows for optimal utilization of resources, reduction in costs, morbidity, and mortality. Mobile ECMO services available “24-7” are needed to provide effective logistics and reliable infrastructure for patient safety. ECMO transports are usually high-risk and complex. To reduce complications during ECMO transport communication using time-out, checklists, and ECMO A-B-C are paramount in any size mobile program. Team members' education, clinical training, and experience are important. For continuing education, regular wet-lab training, and simulation practices in teams increase performance and confidence. In the future the artificial placenta for the extremely premature infant (23–28 gestational weeks) will be introduced. This will enforce the development and adaptation of ECMO devices and materials for increased biocompatibility to manage the high-risk prem-ECMO (28–34 weeks) patients. These methods will likely first be introduced at a few high-volume neonatal ECMO centers. The ECMO team brings bedside competence for assessment, cannulation, and commencement of therapy, followed by a safe transport to an experienced ECMO center. How transport algorithms for the artificial placentae will affect mobile ECMO is unclear. ECMO transport services in the newborn should firstly be an out-reach service led and provided by ELSO member centers that continuously report transport data to an expansion of the ELSO Registry to include transport quality follow-up and research. For future development and improvement follow-up and sharing of data are important.

## Introduction

At the dawn of extracorporeal membrane oxygenation (ECMO) in the 1970s the neonatal population was the first group acknowledged to benefit from this new organ support (1). The number of hospitals which offered ECMO treatment was limited and the risk of transporting neonates on conventional respiratory support was considered high (2, 3). Thus, in 1975 the alternative to transport the patient on conventional critical care support, i.e., to initiate ECMO at the referring hospital before transporting the patient was performed in a neonate (4). Subsequently, the feasibility of ECMO transports has been repeatedly confirmed (5–7).

Concerning most aspects of ECMO outcome (patient safety, resource utilization, quality, morbidity, mortality), there is concensus that ECMO is best provided at high-volume ECMO centers (8–10). However, case mix may influence survival data (11), and Bailly et al. found no association between center size and outcome (12). As recently as a decade ago, only a small number of centers worldwide provided mobile ECMO services for bedside assessment and cannulae insertion. After stabilization, the patient was transported on ECMO for continued support at an ECMO center (13, 14). A transport preceded by bedside assessment, decision, and cannulation for ECMO by direct involvement of the transport team is defined as a *primary transport* (14, 15). A *secondary transport* is a transfer of a patient already on ECMO, often for a day or more, i.e., the mobile team was not directly involved in the cannulation procedure.

In the last decades the numbers of neonatal and pediatric ECMO cases have leveled off or tended to decrease for certain diagnoses (16, 17). In adults, the volume of respiratory and cardiac ECMO treatments and number of ECMO centers are increasing (18). These “young” units gain experience over time albeit the annual treatment volume is unlikely to qualify them as high-volume centers (>20–30 respiratory runs per year) (10). In the future, however, these units may serve as support centers in larger clinical ECMO networks. One example of this, the *Hub-and-Spoke* model, has already been implemented in various health care systems (14, 19, 20). For these to be effective, a “24-7” on-call transport service is needed that provides both primary and secondary transports. Note, a network need not be restrained by national borders for certain diagnoses, i.e., congenital diaphragmatic hernia (CDH), or by a limited population too low to support a low-incidence high-cost therapy. Transports of neonates and children have been described in both national networks (14, 21, 22) and in networks transcending national borders (13, 23, 24).

During the H1N1 pandemic the need for mobile ECMO became evident. However, the medical community has lost control over the number of centers with transport capabilities and the quantity and quality of transports performed. Most importantly, we need to know more about transport related adverse events, how these should be best managed or avoided, and how they correlate to short- and long-term morbidity and mortality outcomes. ECMO transports are unregulated in most countries (14), and authorized transport programs are sparse. Only a handful of publications are based on large numbers of transports (13, 24–26). Even fewer have reported complications during transport. Despite the ELSO transport guidelines (15), an international standard concerning transport management, definitions on adverse events and follow-up are lacking (14). In mixed populations transports adverse events vary from “zero” to >30% (24, 25, 27, 28). In neonates, transport complications may reach as high as 40% as reported by Burgos et al. (23). This high number may coincide with case mix as well as a high frequency of venoarterial ECMO patients and fixed wing transport, both associated with an increased risk for transport complications (24). In a mixed group of both neonatal and pediatric patients five adverse events were reported in 20 ECMO transports (21). In an attempt to identify adverse events a four-level risk category scale was introduced by Ericsson et al. (29), and a revision recently published (24). Death during transport is reported to be rare, <0.5% (14, 24, 25). With no international registry and low published numbers, robust data on mortality is lacking. ECMO transport seems to be safe, at least in the hands of experienced teams. Besides the publications from a few high-volume mobile ECMO programs, additional data is published as cases series or case reports. In a review of 27 case series compared to all ELSO Registry patients, Bryner et al. (25) found no difference in survival for patients transported when stratified for age or ECMO indication. Similar survival results comparing ECMO retrievals and non-transported ECMO cases have been reported from single centers (13, 27).

The aim of this work is to elucidate different approaches to the future development and role for mobile ECMO in the neonatal patient population.

## Discussion

### Where Are We Now?

The decreasing number of ECMO cases in neonates (16) may be attributed to improved technologies and experience in invasive ventilation support, e.g., inhaled nitric oxide, high frequency ventilation, percussive ventilation, etc. It may also be influenced by subtle changes in antenatal care and intervention, e.g., intrauterine procedures such as bronchial blockers used in lung hypoplasia/CDH (30, 31).

The current expansion of adult respiratory and cardiac ECMO, which may see further growth if extracorporeal cardiopulmonary resuscitation becomes well established, not only brings resources but also spread knowledge and awareness concerning the utilization of extracorporeal support in all age groups. Thus, even though referrals for ECMO mainly occur in adult patients, this may also benefit neonates and pediatric patients. An adult center may, depending on local surgeons' training and skills, and hospitals' pediatric/neonatal critical care experiences, provide rescue for a rapidly deteriorating critically ill child. The child is secured, a mobile ECMO team retrieves the patient to an appropriate ECMO center. However, contemporary regional resource utilization may redirect such secondary transport to another region's neonatal or pediatric ECMO center. Adult and pediatric mobile ECMO programs may work in parallel. For example, in the United Kingdom (67 million population) one center, Glenfield Hospital, Leicester, performs all ECMO transports of children, whereas five centers perform regionalized adult transfers (14).

*Safe transport—*the following applies to any size ECMO transport program.

#### Training and Education

The basic training and experience required to become a member of a transport team varies between centers and countries (14). Familiarity with transport equipment used and what differs from the devices used in the ward (14, 24). Guidelines for basic ECMO training and the requirements for team members are published and updated by ELSO (15, 32). Regular training for team members should be arranged with scenarios led by a senior staff. Each scenario is followed by a short discussion. “Water-drills,” using a saline primed ECMO circuit are easily organized, may be performed in small groups and offer opportunities to become familiar with equipment and to train separate procedures (33, 34). Water-drills are excellent to mimic situations in narrow spaces (e.g., elevator, aircraft, etc.). Full high-fidelity simulator training is resource demanding and one complete team is taken from clinical duty for half to an entire day. If more time is allowed, such day may start with a few lectures. However, realistic scenarios are extremely valuable to all team members. Closed loop communication and clear leadership often prove to be what separates the well-performing from the less well-performing team. One to two simulation days per staff per year at centers with >10 treatments/year, and more often in centers of <5–10 treatments/year would be reasonable (33, 35, 36). Team composition, organization of transport program, funding, etc. are not the scope of this work but can be found in the literature (14, 15).

#### Preparing for Transport

(Given that the patient is stable enough for transport.) Infusion lines, ECMO and ventilator tubing, cables, oxygen bottles, etc. are checked, fastened, and secured accordingly. Emergency equipment, i.e., an emergency box (saline, antiseptics, sterile clamps, and scissors, connectors, syringes, 3-way stopcocks), rescue kit (dry oxygenator and centrifugal pump connected with tubing ready for priming with saline), console and drive-unit, as well as blood products are controlled according to a checklist. This checklist ensures confidence of availability of all emergency equipment at “arm's length reach” from the patient. Before un-plugging from the ward a timeout is performed. In this, Situation-Background-Assessment-Recommendation (SBAR) is a suitable structured format (37, 38). Information about “red flags” is important, e.g., circuit clots, earlier bleeding site, etc. Checklist and timeout should be utilized before leaving any location, e.g., ward, CT, operating room, or vehicle (15, 39, 40).

#### On Transport

The timeout is extremely important for safe management when personnel unfamiliar with ECMO are asked to contribute outside their usual comfort zone. An example is when airport staff assists in a patient transfer between transport vehicles. Every step should be explained, and a clear back-up plan has to be known by all participants. The SBAR would be prolonged.

Continuous re-evaluation of the patient follows the classic A-B-C. The *ECMO A-B-C*, displayed in Table 1, focuses on ECMO gear and performance in a structured way and may be used:

1. In emergencies for effective and fast problem solving.
2. For the continuous re-evaluation of patient treatment.
3. In everyday practice as part of ECMO circuit and patient survey at beginning of each shift.
4. After every device or patient related intervention, i.e., if the patient has been moved from one bed to another, if equipment has been changed, etc.
5. After change from one power and/or oxygen source to another, e.g., when the patient has been “un-plugged” on the ward and now relies on batteries and gas bottles, as well as after “plug-in” in ambulance/aircraft, etc.

**Table 1:** Shows an *ECMO A-B-C* to be used for problem solving in emergencies and for routine evaluation of device performance and function in extracorporeal membrane oxygenation support.

**The ECMO A-B-C includes the following items:**
ECMO pump Power/electricity on Revolutions per minute (rpm)—is the pump running and, at correct speed? Flow—does the pump rpm create an adequate ECMO blood flow? Pressures—does the pump produce a pressure adequate for flow and are the pressures obtained reasonable? Trends? Pressures are monitored before (pre-pump pressure), between pump and oxygenator (pre-oxygenator pressure), and in the return tubing back to the patient (post-oxygenator pressure) Sweep gas Flow—sweep-gas flow correctly adjusted? Pressure—is there a pressure in the gas-line to the oxygenator? (indicates integrity of line) Plugged to wall or gas bottle/s? Amount of gas in bottle? Heater on—Power/electricity. There is always risk of hypothermia in the smaller patients, even indoors. Tubing should be lukewarm Tubing Look—the color is an indicator for oxygenation of the blood (darker for venous, bright red for arterial). On transport and in poor lighting conditions a flashlight may be handy for inspection Feel—tubing lukewarm, otherwise check the heater. Chattering of the tubing indicates a drainage problem Cannulation site/s: bleeding? Integrity of distal perfusion line?

*Use your eyes and hands to assess the patient during transport. In aircraft, lighting conditions are often poor (use flashlight!), and the environment noisy. Noise reduction/hearing protection aids should be provided for the patient*.

For all staff to use same robust algorithm, applicable to any occasion, increases confidence and safety for and around the patient.

To reduce complications in neonatal transports, data available today tell us to keep transport time short (24, 29) and to acknowledge that fixed wing (FW) aircraft transports carry a higher risk than ground ambulance. Concerning patient safety, it is important to *get to* the patient as fast as possible (15). For shorter distances <650–800 km, a rapid response concept would be to use helicopter (rotating wing, RW). The mobile ECMO team may dispatch and land at the referring hospital's roof or close nearby reaching the patient bedside much faster than in any ground ambulance and/or FW combination.

When ECMO has been commenced there is more time to consider transport options. The choice of transport vehicle has to be put in its full context as should associated complications. For transports >650–800 km the only feasible mode of transport to keep transport time down would be FW. The most likely contributors to the increased risk observed in FW are longer time on transport and two additional patient movements between transport vehicles. In these procedures, focus may be diverted from patient monitoring and thermoregulation to more practical issues. If staff is aware of which complications are to be expected in the different phases of a transport, numbers may be reduced.

**Figure 1:**
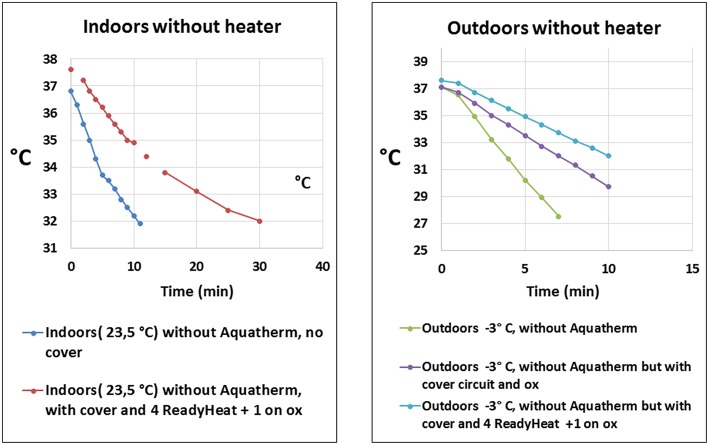
Displays the temperature drop vs. time when simulating a transport of a 3 kg newborn on extracorporeal membrane oxygenation without activated heater (blood warmer). Different means of passive or active protection against hypothermia was used. The left panel shows patient core temperature during movement indoors at an ambient temperature of 23.5°C without activated heater. The right panel shows patient core temperature during transport outdoors at an air temperature of −3°C. With permission from A. Ericsson and C.J. Westlund, ECMO Center Karolinska, Karolinska University Hospital, Stockholm, Sweden (2019). ox, membrane oxygenator. Aquatherm: heater, HICO-AQUATHERM 660; Hirtz & Co., Cologne, Germany. ReadyHeat: Ready-Heat™, disposable self-warming blanket, TechTrade LLC, Jersey City, NJ,USA.

Heat losses and lack of heat conservation are known problems during transports and awareness concerning these problems are important for safe transports. Experiences from transports of neonates show that these patients are at risk of accidental hypothermia (23, 24), and heaters should always be used. During movement of the patient between transport vehicles or from the ambulance to the ward the heater cannot be operated unless an uninterruptible power supply (UPS) is used. However, very few transport programs use UPS (14). In an *ex-vivo* simulation in a mock of a 3 kg newborn on body temperature presented by Ericsson and Westlund at the *35*^*th*^ *Annual CNMS: ECMO* & *the Advanced Therapies for Cardiovascular and Respiratory Failure, 2019, Keystone, CO, USA*, it was not only shown that hypothermia was a risk in out-door transport but also during in-hospital transports, Figure 1. In future designs of transport devices heat loss due to convection and conduction should be taken seriously and prioritized. Future mobile ECMO may expand into transporting smaller patients, and the smaller the patient, the higher the risk for hypothermia. The importance of an ECMO A-B-C (and checklist, SBAR) cannot be emphasized enough to promote a high level of safety.

Even though ECMO in its early development centered on neonates, devices available today are in many cases developed for adults. Centrifugal pumps, for example, are with few exceptions developed for full-flow ECMO in the adult. When used in neonates these pumps may be too coarse in their flow dynamics for safe use. A centrifugal pump running at low flow speed may induce hemolysis and platelet/coagulation activation due to long residence for platelets and red blood cells inside the pump (and other circuit components) (41). Proper sizing of pump devices will reduce the risk of hemolysis and coagulation activation, thus also bleeding complications. Effective integrated heaters are important for safe transport of the newborn. With miniaturized implantable gas-exchangers the need for heaters may decrease in the future. These products, however, are not likely to be seen in neonates initially but rather in chronic adult patients bridged for transplant, etc.

### Where Will We Go?

Today the number of neonatal ECMO treatments has become rather constant in most of the developed world. Thus, the likelihood of seeing an increase in the number of neonatal ECMO transports with conventional diagnoses and established criteria for ECMO support is low. Socioeconomic and other factors slow or inhibit the extension of major ECMO programs. New methods for extracorporeal life support for the premature are in development. The artificial placenta (AP) focuses on support in the extremely premature (23–28 weeks gestational age, GA) (42–44). To predict the volume of extremely prematurely born infants to be offered AP, or the spread of this life support mode and the extent of engagement by mobile ECMO teams is impossible. First clinical trials will likely start within 5 years (45). However, this may occur sooner as single center studies in humans are planned in the near future to be followed by multicenter approaches, and the method may be commercially available in the not too distant future (*personal communication: Professor Alan W. Flake, Center for Fetal Research, Department of Surgery, Children's Hospital of Philadelphia, Philadelphia, PA, USA*). A major and perhaps unforeseen impact of the AP is that it will elicit an ethical debate where public opinion and media pressure will enforce research concerning *prem-ECMO*. Prem-ECMO is support in the prematurely born GA 28–34 weeks who are “too old for the AP,” but still “too young for conventional ECMO” (46, 47). Today these infants are denied ECMO due to the high risk of cerebral bleeding complications. Improvements in design are centered on coating/lining materials, pumps, gas exchangers, and cannulae. However, insights in the management of anticoagulation as well as ventilation strategies are important. The ventilated lung (and inotropes) may be part of the pathophysiology of cerebral bleedings in the preterm (48, 49). Concerning prem-ECMO transport, it could start tomorrow—the infrastructure is already available by caring for the GA 34+ weeks children.

If, or rather when, prem-ECMO transport is launched, it seems clear that these transports will include high risk patients providing new challenges. Even given that we have proper devices the risk of hypothermia remains. Smaller patient not only requires thinner cannulae, but the margin for error in placement will be small and risk of dislodgement considerable. The implementation of prem-transport has to be guided by adequate protocols and be evaluated. Today we are far beyond the time when anecdotes can mark the path to be followed.

In this article, the impact of stem cell/gene therapy in the neonate will not be discussed and what the future holds is yet to be seen (50). However, AP patients have been suggested as one group for gene therapy (44).

In ECMO transports a lowest acceptable number, or minimum of total accumulated annual transport hours required to ensure patient safety has not been published. However, it may be assumed that the larger the patient volume the better the outcome with reduced morbidity and mortality. The first step needed for us as a community would be to agree on standards, acknowledge that adverse event do occur in any mobile ECMO program and from this create a platform to improve and develop our programs. Resources should be allocated to expand the ELSO Registry with a transport module for reporting but also for extraction of own in- and processed out-put data. ELSO Centers of Excellence with recognized transport programs could be encouraged to take the lead in the development and support of interhospital mobile ECMO and act as “role-models” for safe and reliable mobile ECMO.

## Conclusions

The expansion of neonatal ECMO into new geographical regions is limited. Hence, future volume increases in mobile ECMO in neonates depend on the introduction of new methods for the (extremely) premature: the artificial placenta (GA 23–28 weeks) and what comes thereafter, prem-ECMO (GA 28–34 weeks).

For safe transport of any age patient and in any size program, basic requirements for education, clinical training and experience are needed. Regular wet-lab training and high-fidelity team simulations using clinical scenarios increase performance. Time-outs, checklists and ECMO A-B-C are paramount for safety in-hospital and on transport. For future development and improvement follow-up and sharing of data is important.

ECMO transport services in the newborn should include an out-reach service provided by ELSO member centers that report transport related data to an expansion of the ELSO Registry for transport quality follow-up and research.

## Data Availability

No datasets were generated or analyzed for this study.

## Author Contributions

The author confirms being the sole contributor of this work and has approved it for publication.

### Conflict of Interest Statement

LB is a Medical Advisory Board member of Eurosets (Mirandola, Modena, Italy), and of Xenios (Heilbronn, Germany).
